# Antibody complementarity determining region design using high-capacity machine learning

**DOI:** 10.1093/bioinformatics/btz895

**Published:** 2019-11-28

**Authors:** Ge Liu, Haoyang Zeng, Jonas Mueller, Brandon Carter, Ziheng Wang, Jonas Schilz, Geraldine Horny, Michael E Birnbaum, Stefan Ewert, David K Gifford

**Affiliations:** 1 MIT Computer Science and Artificial Intelligence Laboratory, Cambridge, MA, USA; 2 Department of Electrical Engineering and Computer Science, Massachusetts Institute of Technology, Cambridge, MA, USA; 3 Novartis Institutes for BioMedical Research, Basel, Switzerland; 4 Department of Biological Engineering, Massachusetts Institute of Technology, Cambridge, MA, USA; 5 Koch Institute for Integrative Cancer Research at MIT, Cambridge, MA, USA

## Abstract

**Motivation:**

The precise targeting of antibodies and other protein therapeutics is required for their proper function and the elimination of deleterious off-target effects. Often the molecular structure of a therapeutic target is unknown and randomized methods are used to design antibodies without a model that relates antibody sequence to desired properties.

**Results:**

Here, we present Ens-Grad, a machine learning method that can design complementarity determining regions of human Immunoglobulin G antibodies with target affinities that are superior to candidates derived from phage display panning experiments. We also demonstrate that machine learning can improve target specificity by the modular composition of models from different experimental campaigns, enabling a new integrative approach to improving target specificity. Our results suggest a new path for the discovery of therapeutic molecules by demonstrating that predictive and differentiable models of antibody binding can be learned from high-throughput experimental data without the need for target structural data.

**Availability and implementation:**

Sequencing data of the phage panning experiment are deposited at NIH’s Sequence Read Archive (SRA) under the accession number SRP158510. We make our code available at https://github.com/gifford-lab/antibody-2019.

**Supplementary information:**

Supplementary data are available at *Bioinformatics* online.

## 1 Introduction

The identification of human antibodies and receptors with high affinity and specificity to human disease-associated targets is a key challenge in producing effective human therapeutics. At present antibody sequences are discovered *in vivo* using animal immunization or by *in vitro* affinity selection of candidates from large synthetic libraries of antibody sequences (Breitling *et al.*, 1991; McCafferty *et al.*, 1990; O’Brien and Aitken, 2004; Scott and Barbas, 2001; Smith, 1985). These methods both have the advantage that they do not require the structure of a target to be known for antibodies to be discovered. However, they are empirical and do not produce a model of sequence space that admits computational optimization and specificity analysis without conducting additional experiments such as counter panning.

Most computational methods for antibody design optimization assume that the structure of a target is known; however, target structure is unknown in many cases (Fischman and Ofran, 2018). Other approaches seek to optimize antibody properties primarily focused on predicting the structural conformation of the CDR-H3 loop, which to date remains a difficult unsolved challenge (Kuroda *et al.*, 2012; Reczko *et al.*, 1995). Many such approaches are based on calculations of binding free energies, where the multitude of possible expressions have been found to be of highly variable quality for predicting actual affinities in experiments (Kunik and Ofran, 2013; Moal *et al.*, 2011). More recently, neural network methods have been successfully applied to predict which region of the CDR will be in contact with an antigen without reliance on structure prediction, but this work does not provide insights on how to obtain improved affinity (Liberis *et al.*, 2018). Existing models of antibody binding do not produce gradients for sequence optimization that directly relate antibody sequence to a desired objective.

Here, we describe the application of high-capacity machine learning to design antibody sequences, rather than predicting their properties. Unlike other computational approaches, the molecular structure of an antigen does not need to be known, permitting our method to be applied to any target used in affinity selections. Our hypothesis is that with sufficient sequence-only training data, high-capacity machine learning can sufficiently model the biophysics of antibody-target interactions to generalize from these observations to produce novel improved antibody sequences. We tested our hypothesis by generating training data that relates Fab fragments with varying CDR-H3 sequences to their enrichment in phage display panning experiments with four target antigens and one mock control. The four targets we focused on in this work include ranibizumab, bevacizumab, etanercept and trastuzumab (Supplementary Data). Our mock control consisted of panning with no antigen and thus measures CDR-H3 sequence specific bias for display on phage and phage propagation.

We first show that neural networks trained on other Fc-region containing targets including trastuzumab and etanercept can predict antibodies that were enriched for bevacizumab but not for the other Fc-region containing targets. This result demonstrates that models learned from multiple targets can be combined and transferred to a new target to select antibodies with desired properties.

**Fig. 1.**
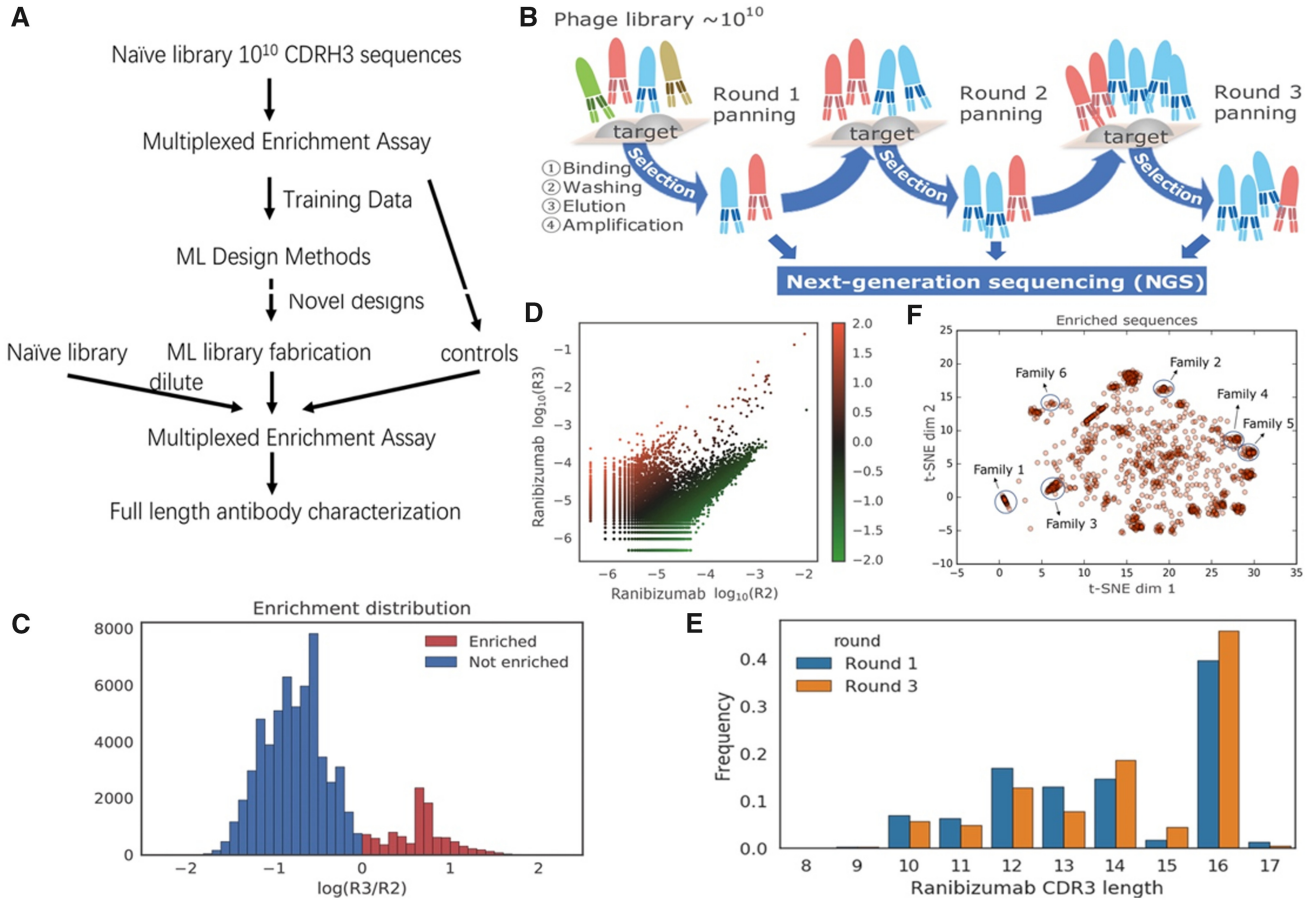
Summary of the training data used in our machine learning framework. (**A**) Diagram of the workflow for antibody optimization. (**B**) Diagram of training data generation using phage display panning and NGS. Three rounds of panning (R1, R2, R3) were performed. We characterize the frequency of CDR-H3 sequences at the ends of each panning round to compute enrichment. (**C**) Histogram of R2-to-R3 enrichment for ranibizumab in training data. *Y*-axis denotes the sequence counts in each bin. (**D**) Scatter plot of log10 sequence frequency in R2 and R3, colored by the R2-to-R3 enrichment value. Each point represents a unique valid sequence in the NGS output, where points above the diagonal have positive R2-to-R3 enrichment and vice versa. (**E**) Histogram of CDR-H3 sequence length before and after two rounds of panning. *Y*-axis denotes the proportion of reads in each bin. Ranibizumab binders (survived sequences in Round 3) tend to exhibit greater CDR-H3 lengths (compared to sequences in Round 1). (**F**) t-SNE visualization of sequences with over 10-fold enrichment between Round 2 and Round 3. Sequences similar to the ones in Table 2 are labeled by circles

We next show that we could design improved antibody sequences with a two-stage approach, which we named *Ens-Grad*, by modeling antibody affinity with an ensemble of neural networks and efficiently optimizing it with gradient-based optimization (Fig. 1A). Ens-Grad produced accurate predictive estimates of affinity enrichment for previously unseen sequences. With a small design budget of 5467 sequences, Ens-Grad produced antibody sequences that were superior to all of the sequences present in our training data. We interpreted our machine learning models by computing the minimal sets of specific CDR-H3 amino acids required for binding (Carter *et al.*, 2019a, b). Our results demonstrate that machine learning can produce useful models of antibody affinity that can create novel sequences with desired properties and increase target specificity.

## 2 Materials and methods

### 2.1 Phage-panning data generation

The experimental details of the initial and follow-up campaigns of phage-panning experiments, including the initial single framework library generation, Immunoglobulin G (IgG) expression, phage display panning against target molecule, next-generation sequencing sample preparation, data preparation, oligo synthesis of CDR-H3 sequences and the follow-up library generation, are described in Supplementary Data.

### 2.2 Training an ensemble of neural networks to predict enrichment from antibody sequence

We used six different architectures in Ens-Grad, five of which were convolutional neural networks with one or two convolutional layers with filter size of 1, 3 or 5 residues and stride 1, followed by a local max-pooling layer with window size 2 and stride 2. We used 64 and 32 convolutional filters for single convolutional layer networks. In one of the double convolutional layer networks, we used 32 filters with width 5 in the first layer and 64 filters with width 5 in the second layer. In the other network, we used 8 convolutional filters with width 1 in the first layer to learn an embedding from one-hot to hidden space for each amino acid, and then used 64 filters with width 5 to learn higher-level patterns. In each of the convolutional neural networks, the output from the last convolutional layer was fed into a fully connected layer with 16 hidden units and a dropout layer. It is then connected to the final output layer where the loss function is evaluated. We also designed a two-layer fully connected neural network with 32 hidden units and dropout in each layer. Table 1 illustrates the detailed setup of each architecture and an estimation of the number of parameters in each architecture. The details of the training procedures are described in the Supplementary Data.

**Table 1.** Neural network architectures used in the Ens-Grad model ensemble and the number of parameters in each neural network model

Name	Number of convolutional layers	Conv 1 (width, #filters)	Conv 2 (width, #filters)	Number of fully connected layers	Number of fully connected neurons	Total number of parameters
Seq_32_32	0	Not applicable	Not applicable	2	32	13 954
Seq_32x1_16	1	Width 5, 32	Not applicable	1	16	8402
Seq_32x2_16	2	Width 5, 32	Width 5, 64	1	16	18 706
Seq_64x1_16	1	Width 5, 64	Not applicable	1	16	16 754
Seq_32x1_16_filt3	1	Width 3, 32	Not applicable	1	16	7122
Seq_embed_32x1_16	2	Width 1, 8	Width 5, 32	1	16	13 082

### 2.3 Optimizing antibody sequences with gradient ascent and a neural network ensemble

**Fig. 2.**
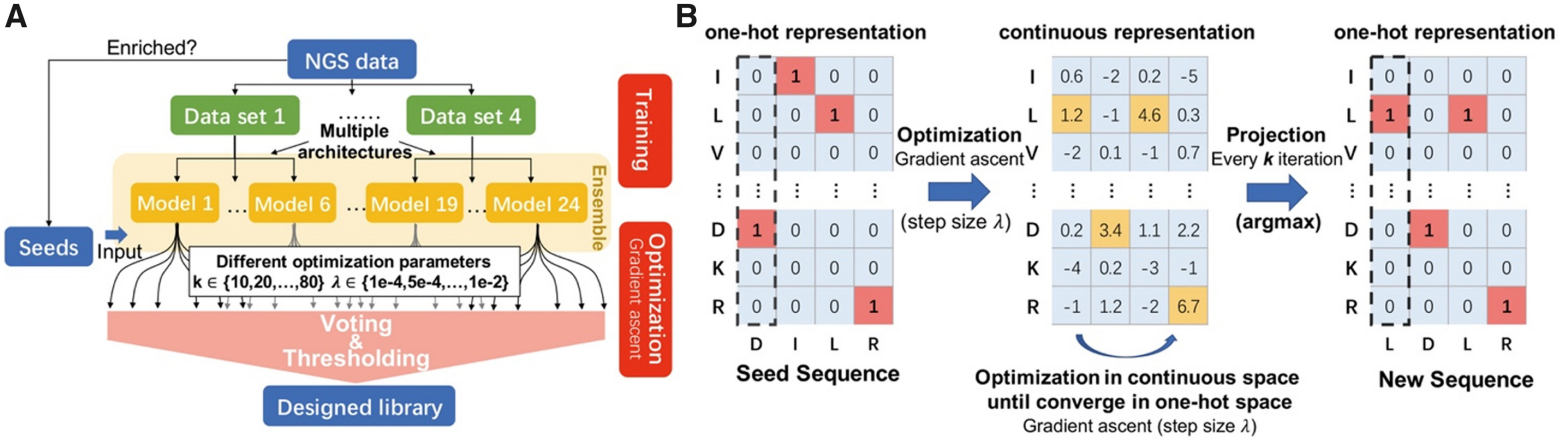
Overview of the Ens-Grad sequence-optimization methodology. (**A**) Neural network ensemble optimization and thresholding pipeline. (**B**) Optimization of a single sequence in one-hot space and continuous space using gradient ascent

In Ens-Grad, we used the gradients back-propagated from the neural network output layer to the input layer to guide the improvement of an input sequence, considering that direct optimization on one-hot discrete space is difficult and inefficient. This use of backpropagation is in contrast to its usual application of updating network parameters as in this application network parameters are held constant. Similar technique has been used for visualization of neural networks (Erhan *et al.*, 2009; Liu *et al.*, 2019; Simonyan *et al.*, 2013). We relaxed the one-hot constraint during optimization to fully take advantage of gradient methods, periodically projecting the current continuous representation back to a discrete one-hot input by taking the argmax of each amino acid position (Fig. 2B). The resulting sequence is then evaluated and compared to the best recorded previously projected sequences from the same seed. If the newly projected sequence fails to improve upon the best score for 10 iterations we terminate the optimization, presuming it has converged to local optimum of the network’s score function in one-hot space. By choosing different step sizes *λ* and projection intervals k, we were able to control the level of divergence between proposed sequences and seed sequences. In addition, we employed ensembles to increase diversity in the optimized sequences and used a two-step voting-thresholding strategy to increase the robustness of our optimization results (Supplementary Fig. S2A).

### 2.4 Alternative machine learning methods for proposing sequences

In addition to our neural network ensemble (Ens-Grad), three other machine learning frameworks were also applied to generate novel CDR-H3 sequences. We describe the details of these methods in the Supplementary Data.

## 3 Results

### 3.1 Machine learning predicts panning enrichment

To select Fab sequences against ranibizumab we conducted three rounds of phage display panning (Fig. 1B). In parallel, we conducted so-called mock panning in which we infected and propagated phages without contact to an antigen. The synthetic input library for panning contained a fixed framework with fixed CDR sequences except for CDR-H3. CDR-H3 was randomized with ∼10^10^ different sequences that ranged in length from 10 to 18 amino acids. We obtained 572 647 unique CDR-H3 sequences for ranibizumab in Round 1 (R1), 297 290 in Round 2 (R2) and 171 568 in Round 3 (R3). We define enrichment as the log10 of the round-to-round ratio of sequence frequencies and R2-to-R3 enrichment was used for training as it had a higher signal-to-noise ratio. Data preprocessing and denoising is described in detail in Supplementary material (Data preparation section). Both directions of enrichment and certain preferred CDR-H3 lengths were observed for ranibizumab (Fig. 1C–E). Moreover, in t-SNE visualization the CDR-H3 sequences with high enrichment form isolated clusters that correspond to distinct enriched sequence families (Fig. 1F, Supplementary Fig. S1).

**Fig. 3.**
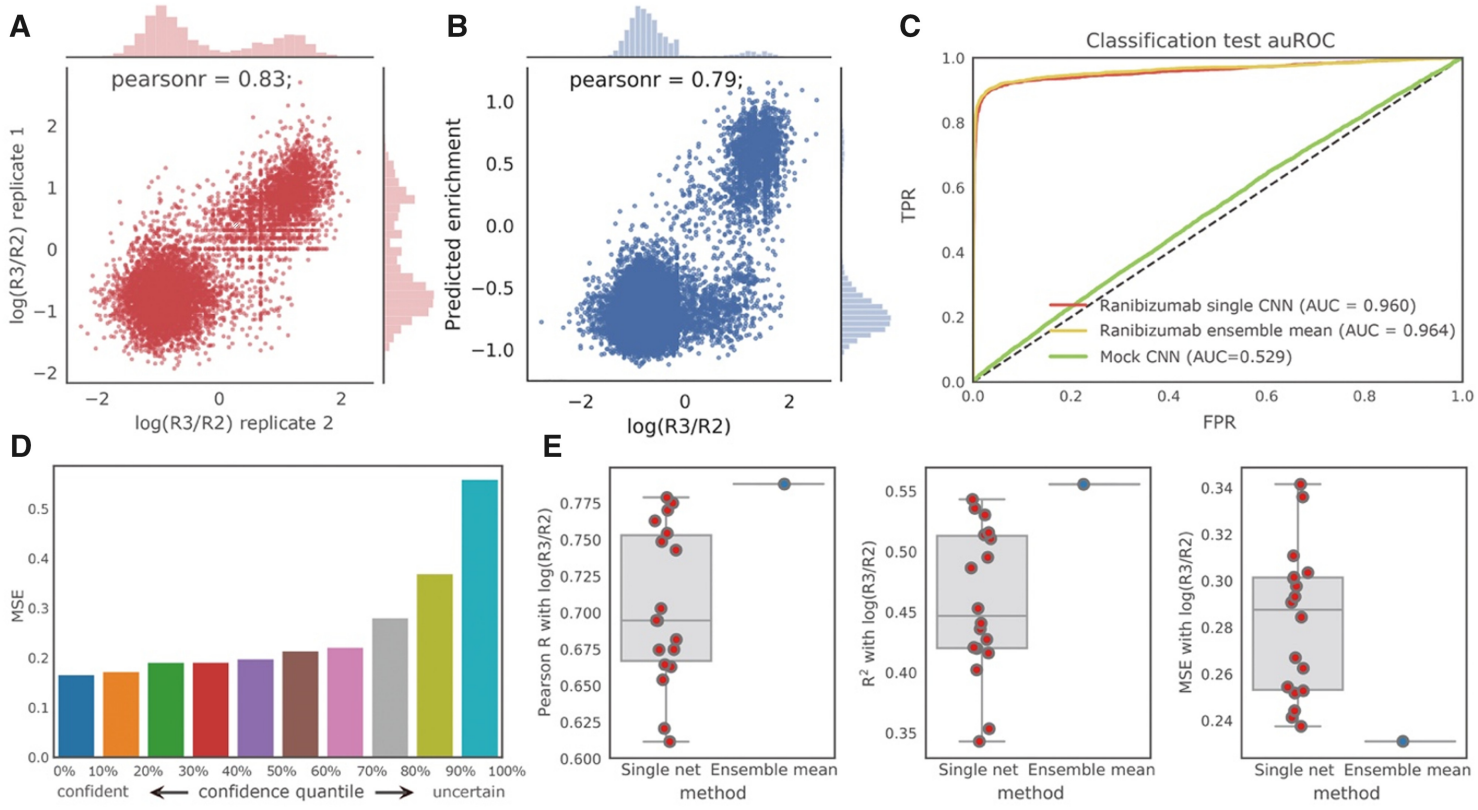
Performance of our neural networks in predicting ranibizumab enrichment. (**A**) Replicate consistency of enrichment for ranibizumab (Pearson *r* = 0.83, *P* < 1e−16, the *P*-value indicates the probability of an uncorrelated datasets having a Pearson correlation at least as extreme as the one computed from this dataset, same below); the Pearson *r* is about the same between replicates as between the neural network predictions and the observed enrichment values, suggesting that our prediction performance is bounded by the replicate consistency. (**B**) Regression performance for ranibizumab on non-overlapping sequences in replicate II using the mean prediction of the ensemble (Pearson *r* = 0.79, *P* < 1e−16). (**C**) ROC curve of classification task for ranibizumab using the best single network and the ensemble, compared with a mock control. (**D**) Uncertainty estimates of ensemble correlate with prediction accuracy. (**E**) Ensembles (blue) produce better estimates of enrichments than any single network (red) in terms of Pearson *r*, *R*^2^ and mean square error

We found that using a convolutional neural network one can accurately predict the R2-to-R3 enrichment of held-out test sequences. We trained our models on replicate I for R3 and tested on the non-overlapping sequences in replicate II. Our model achieved an AUROC (area under the receiver operating curve) of 0.960 and a Pearson *r* of 0.79 (Fig. 3B and C). The direction of enrichment (positive or negative) was used for classification and the real-valued observed R2-to-R3 enrichment was used for regression. Given a 0.83 correlation (Pearson *r*) between two biological replicates (Fig. 3A), we expect model performance is bounded by experimental noise. When trained and tested on the mock control with a random 4:1 train–test split, an AUROC of 0.529 and a Pearson *r* of 0.025 were observed, consistent with our expectation of no predictive ability when attempting to infer phage enrichment in the absence of target binding.

To test the model’s capability to extrapolate beyond best seen sequences, we built an independent held-out validation set consisting of top 4% of the all observed sequences using R2-to-R3 enrichment. We trained a convolutional neural network for regression task on the lower 96% sequences, and compared the predicted scores between the held-out top sequences and top 4% of training sequences. Our model assigned higher scores to the unseen sequences when compared with top seen sequences, indicating that convolutional neural network is able to extrapolate beyond seen examples. We also observed that the network was able to assign higher score to the upper half of the held-out set when compared with the lower half (Supplementary Fig. S2).

### 3.2 Ensembles of neural networks improve accuracy and characterize uncertainty

A method that produces novel antibody sequences will naturally need to venture in sequence space outside of observed training data. Thus, when evaluating sequences, it is important to estimate both the expected value of enrichment as well as the model uncertainty of the estimated expectations. Employing ensemble learning (Carney *et al.*, 1999; Lakshminarayanan *et al.*, 2017; Zhou *et al.*, 2002), we accomplish this with an ensemble of 18 neural network models of six architectures that have been trained on three different sets of observations for each architecture (Section 2).

We first asked whether the uncertainty encoded in the ensemble correlates with prediction accuracy. We quantified the prediction accuracy using mean square error (MSE) and estimated ensemble uncertainty by computing the variance of the predictions of the models in the ensemble. With the ensemble trained on panning replicate I, we evaluated the uncertainty and accuracy on sequences only observed in replicate II. We observed a strong positive correlation between the MSE and the variance of the ensemble prediction (Fig. 3D), showing that confident predictions from the ensemble are more accurate. This indicates that our ensemble model is well-calibrated.

We hypothesized that the mean of an ensemble of models would result in a more robust estimator of sequence enrichment than using prediction from a single network. We compared the performance of our 18 single models with an ensemble method that averaged the prediction of all 18 models. Evaluating on our held-out data, we observed that the ensemble model outperformed all of our 18 models in all metrics considered including Pearson *r*, *R*^2^ and MSE (Fig. 3E).

### 3.3 Machine learning can eliminate non-specific antibodies that bind to undesired targets

**Fig. 4.**
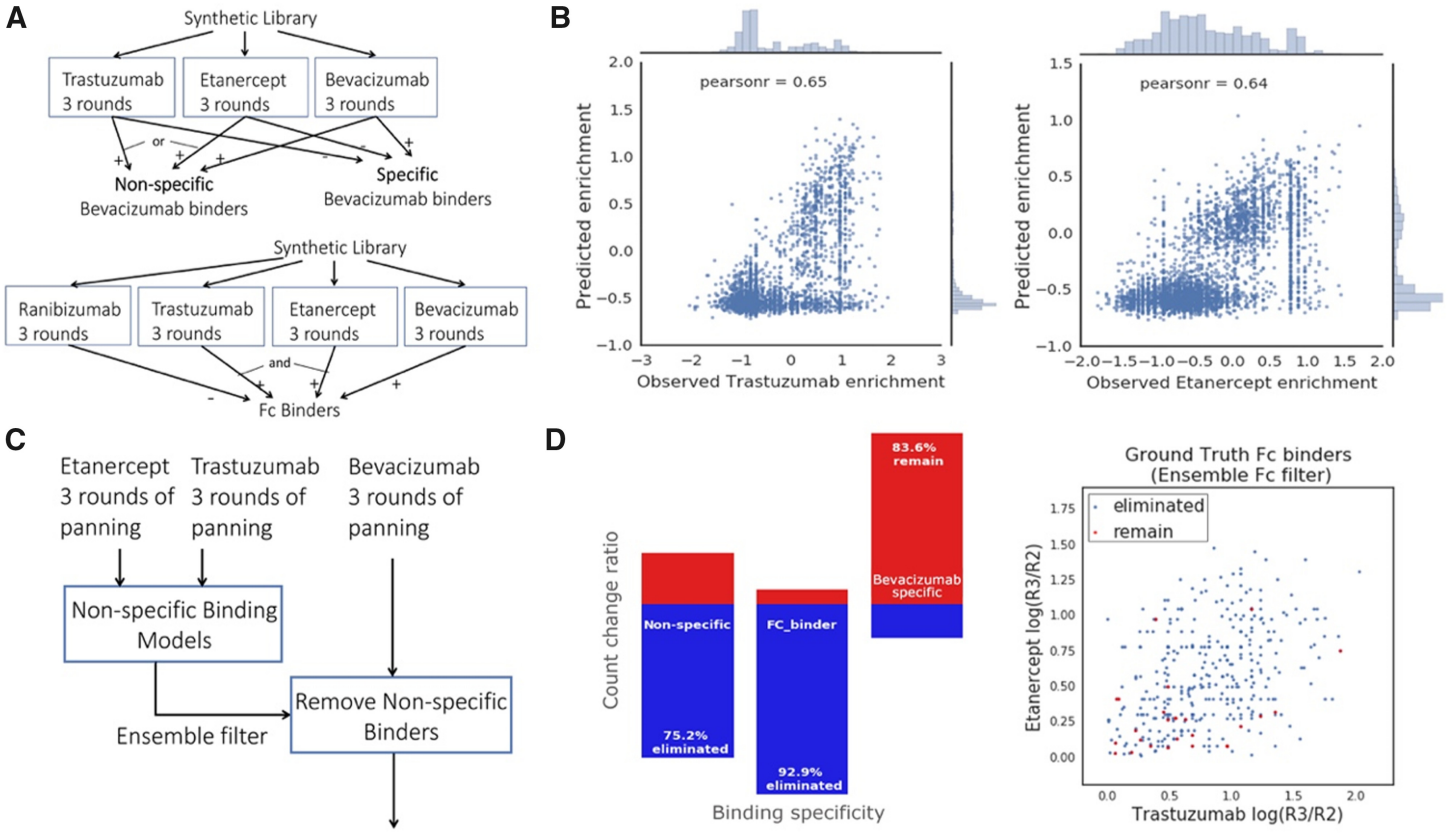
Machine learning can reject undesired antibodies. (**A**) Definition of non-specific, specific bevacizumab binders and Fc binders using panning experimental data. Positive sign represents positive R2-to-R3 enrichment and negative sign represents non-positive enrichment. (**B**) Regression performance of a multi-output network on a randomly held-out 5% test set with no-missing values for trastuzumab (left, Pearson *r* = 0.65, *P* < 1e−16) and for etanercept (right, Pearson *r* = 0.64, *P* < 1e−16). (**C**) Diagram of the non-specific binding prediction task. (**D**) Changes in count of non-specific antibodies, FC-binders and bevacizumab-specific antibodies after filtering antibodies that were enriched for bevacizumab using the specificity prediction ensemble (left). Bevacizumab binding antibodies plotted vs. their etanercept and trastuzumab enrichment, both of which contain an Fc region (right). Blue dots represent ground truth bevacizumab Fc binders that were eliminated and red dots represent Fc binders remained after applying the corresponding filter on all bevacizumab binders

We hypothesized we could create a model to identify antibodies that bind etanercept or trastuzumab, and then use this model to reject anti-bevacizumab antibodies that bind to etanercept, trastuzumab, or the IgG Fc region they share with bevacizumab. To this end, in addition to the previously described ranibizumab panning experiment, we further conducted three rounds of phage display panning (Fig. 1B) separately against etanercept, trastuzumab and bevacizumab starting with the same randomized library. We defined the ground-truth specific/non-specific bevacizumab binders and Fc binders by whether one has positive R2-to-R3 enrichment in the panning experiments against each target (Fig. 4A).

We trained an ensemble of six multi-output neural networks that output predicted R2-to-R3 enrichment of both etanercept and trastuzumab (Fig. 4A;Table 1; Section 2). We randomly held out 50% of the non-specific bevacizumab binders that also appeared in the etanercept and trastuzumab experiments from the training set. We calculated the ensemble variance to incorporate model uncertainty, and used the 95% confidence upper bound of the predicted scores for trastuzumab and etanercept to indicate potential non-specific binding.

By removing bevacizumab binders that had a positive confidence upper bound score for either etanercept or trastuzumab (Fig. 4C), our non-specific prediction ensemble successfully eliminated 75.2% of general non-specific binding to either trastuzumab or etanercept (Fig. 4D, Supplementary Table S1, *P* < 1e−16). We found that the percentage of bevacizumab-specific antibodies was increased by 7.4%, and 83.6% of the bevacizumab-specific antibodies remained after filtering (Fig. 4D, Supplementary Table S1), showing that the procedure successfully reduces non-specific binding while retaining most target-specific binding.

We defined 366 sequences as ground truth IgG Fc binders that showed positive R2-to-R3 enrichment in bevacizumab, etanercept and trastuzumab (all Fc containing) but non-positive enrichment in ranibizumab (no Fc region). Our non-specific prediction ensemble successfully eliminated 340 of the 366 Fc binders (92.9%) and the remaining sequences have low R2-to-R3 enrichment (Fig. 4D, *P* < 1e−16).

### 3.4 Machine learning designed sequences are better than panning derived sequences

Our next aim was to examine whether computational models are capable of designing previously unseen CDR-H3 sequences with improved affinity for ranibizumab (Fig. 1A). We employed a gradient-based optimization framework (Ens-Grad) to propose high-affinity sequences from an ensemble of neural networks trained to predict enrichment. For comparison, we also applied alternative computational approaches to propose improved sequences including a variational autoencoder (VAE) and genetic algorithms (GA-KNN, GA-CNN; Section 2). We selected sequences with either non-negative R2-to-R3 enrichment or larger than 5e−5 frequency in R3 as seed sequences that were used to initialize the optimization for proposing novel sequences. Both the seeds and sequences that the neural networks predicted to be negative were subsequently included as experimental controls to validate our computational methods and to provide baseline performance. Collectively we constructed a library of 104 525 sequences for experimental testing (Section 2).

**Fig. 5.**
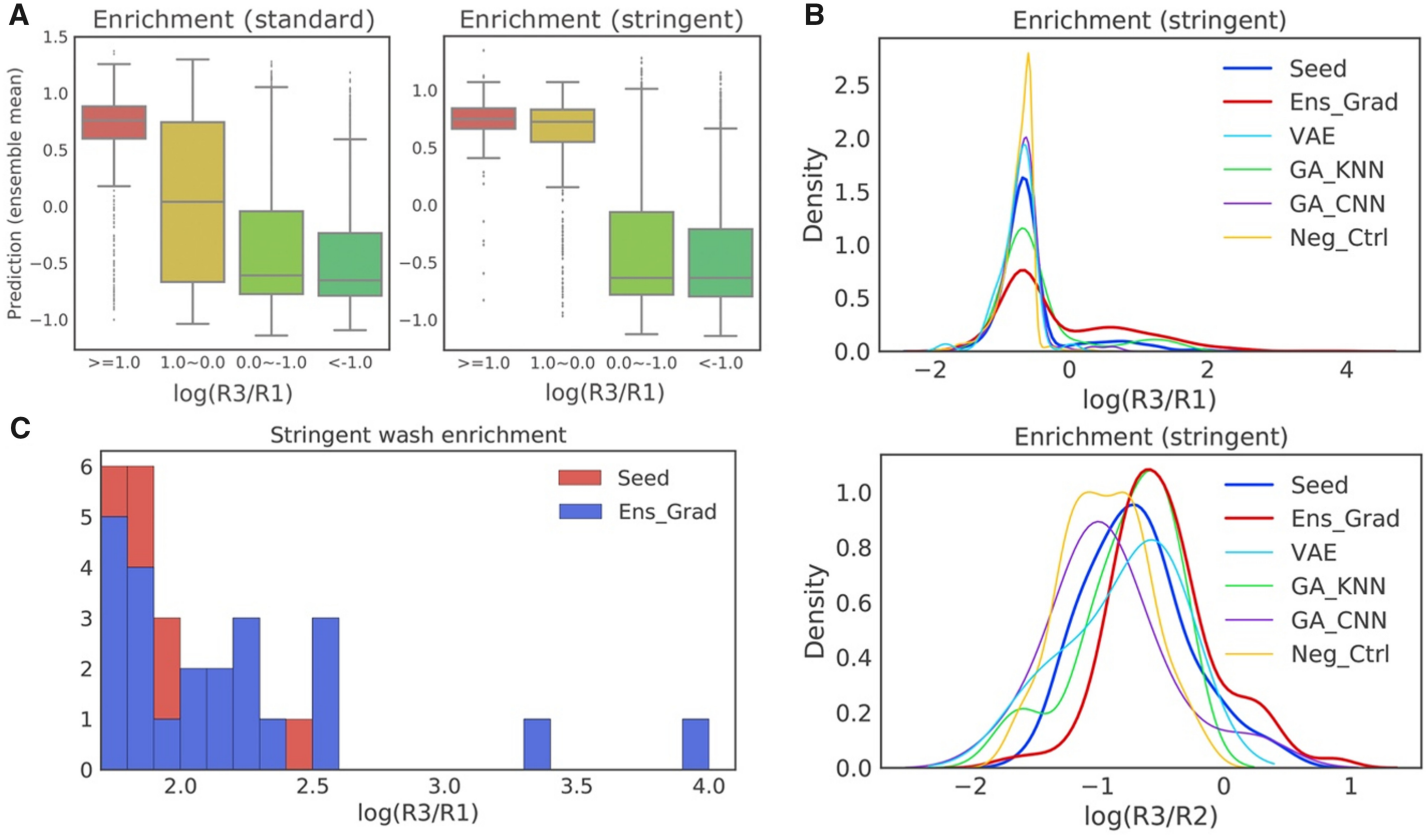
Experimental results of the ML-proposed library diluted 1:100 into our initial synthetic library. (**A**) Predicted enrichment using ensemble of all sequences in the synthesized library, including optimal sequences, seed sequences and negative controls, grouped by their experimental R1-to-R3 enrichment. Ens_Grad predicts higher values for groups with higher experimental binding affinity. (**B**) Distribution of R1-to-R3 and R2-to-R3 enrichment of sequences proposed by each of the methods, together with seed sequences and negative controls. Ens-Grad proposed sequences that are better in general by introducing a positive distributional shift compared to seeds, whereas the other methods failed to improve upon the seed sequences. (**C**) Stacked histogram of the right tail of R1-to-R3 enrichment distribution of each method. Ens-Grad sequences were in higher percentage on the right tail, and the best Ens-Grad sequence outperformed the best seed sequences by 28-fold. None of the sequences designed by other methods attained enrichment higher than 0.638 and thus do not appear in this plot

We experimentally tested the synthetic sequences in a three-round phage display panning experiment using both stringent and standard washing conditions (Supplementary Data). We cloned the designed library into a phage Fab expression framework with the other CDR sequences fixed to their values during training data generation, and created phage that displayed this library. This phage library was diluted 1:100 into a synthetic library of complexity ∼10^10^, such that the library complexity was equivalent to the previous experiment and our designed sequences were not underrepresented. We used the 1:100 library in a three-round phage display panning experiment and analyzed the R1-to-R3 enrichment which reflects the full spectrum of binding affinity. Evaluated on all of the tested sequences, including controls and optimized sequences, we found that the observed R1-to-R3 enrichment correlates well with the prediction from our ensemble of neural networks (Fig. 5A).

We compared the enrichment of the computationally optimized sequences and the seeds in the three-round phage display panning experiment. We observed that the 5467 sequences proposed by Ens-Grad show a positive distributional shift of R1-to-R3 enrichment compared to seed sequences (Fig. 5B; two-sided Mann–Whitney U test; for standard washing condition, *P* = 5.324e−13; for stringent washing condition, *P* = 1.333e−13). With stringent washing, the mean R1-to-R3 enrichment value of Ens-Grad derived sequences is 0.341 log10 higher than that of the seeds, indicating an improvement equivalent to 62.13% of the standard deviation of seeds R1-to-R3 enrichment (0.549). The Ens-Grad method also produced the sequences with the highest enrichment at the upper tail of the enrichment distribution (Fig. 5C), outperforming all top sequences in seeds by 28-fold (1.450 in log10 fold). None of the other computational methods produce sequences with R1-to-R3 enrichment higher than 0.638 (log10 fold). Ens-Grad produced sequences with up to five changes away from seeds, with certain sequences with two changes producing sequences with more than a hundredfold R1-to-R3 enrichments (Supplementary Fig. S3C). We observed negative or insignificant positive distributional shift (two-sided Mann–Whitney U test; *P* = 0.025 for VAE, *P* = 0.140 for GA-CNN, *P* = 0.776 for GA-KNN) of R1-to-R3 enrichment in sequences produced by each of the three alternative baseline methods. Similar patterns were observed for the distribution of R2-to-R3 enrichment.

**Fig. 6.**
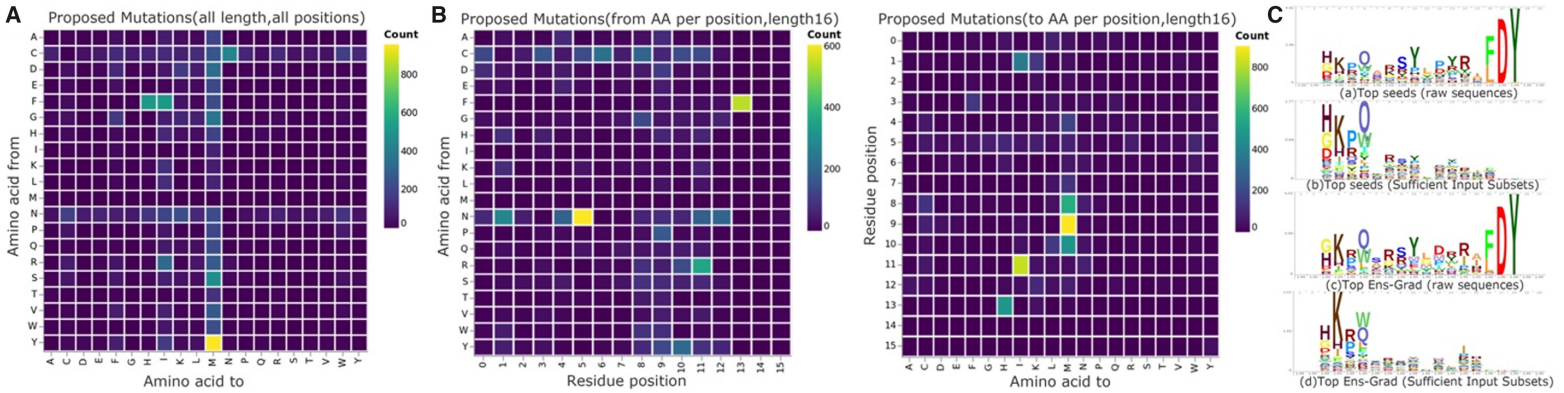
Visualization of machine learning (ML) optimization. (**A**) Position independent proposed mutations map. Heatmap visualization showing the count of observed sequence mutations between seed and corresponding ML-proposed sequences. The mutation count indicates the number of mutations observed from/to each amino acid in 7614 pairs of seed and ML-proposed sequences, regardless of the position of the mutated residue. Cysteine and asparagine are not proposed by our ML method (Section 2). (**B**) Position dependent proposed mutations map (sequences length 16 only). Heatmap visualization showing the count of ML-proposed sequence mutations occurring from (left) and to (right) each amino acid at each sequence position. The mutation count indicates the number of mutations observed from/to each amino acid at each sequence position in 5025 pairs of seed and ML-proposed sequences having length 16). (**C**) Sequence logo visualizations for top 1000 sequences of length 16 based on mean predicted R3/R1 enrichment from the neural network ensemble. Sequence logos for Seeds (a) and ML-proposed (c) groups are based on residue frequency, and sequence logos for Seeds (b) and ML-proposed (d) are based on frequency of residues marked as most important using Sufficient Input Subset for predicted sequence enrichment. Sequence logos are computed using Skylign

We selected a subset of the highly enriched sequences including 12 seeds and 7 machine learning-designed sequences from six different sequence families and synthesized complete IgG molecules of these sequences to evaluate their affinity using an ELISA EC50 assay (Supplementary Data). Within each CDR-H3 sequence family, we observed that the machine learning-designed sequences had superior or equivalent affinity for ranibizumab than any of the seed sequences, with the best machine learning-designed antibody having an EC50 of 0.29 nM (Table 2, Supplementary Fig. S3B). We found that a subset of CDR-H3 amino acid positions and values were Sufficient Input Subsets (Carter *et al.*, 2019a, b) for Ens-Grad to predict enrichment at a threshold of 0.4 (Table 2) 17. We also found Ens-Grad preferred to change certain CDR-H3 amino acid positions to optimize enrichment, and the Ens-Grad Sufficient Input Subsets necessary for enrichment diverged from the simple observed frequency of optimized sequences (Fig. 6).

**Table 2.** Examples of the top ML-proposed sequences and top seed sequences, along with their enrichment in standard and stringent washing condition, EC50 affinity measurement and R2 fit of EC50 model

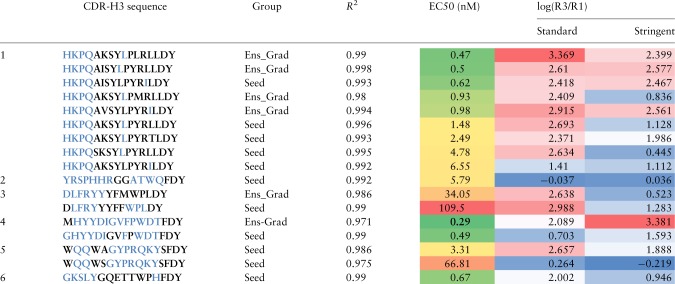

*Note:* See Supplementary data for how these sequences are chosen. Sequence positions colored in blue comprise a Sufficient Input Subset, which alone provides sufficient evidence for the ensemble to justify a highly positive prediction ≥0.4 of enrichment. The color shades for EC50 uses a 3-color scale where the max value is colored red, min value is colored green, median is colored yellow and the rest are colored proportionally. The log(R3/R1) column is colored with 3-colored scale where max value is colored in red, min value is colored in blue and median is colored in white.

Column 1 is the family number of the sequence (see Fig. 1F).

## 4 Discussion

We have found that machine learning-based methods are an effective way to both model and optimize antibody complementarity-determining region sequences based upon experimental training data. We note that our methods are not intended as an alternative to conventional randomized affinity maturation that can explore tens of millions of alternative candidates. Rather, machine learning provides a modular method to combine models based on data from both primary antibody campaigns and subsequent affinity maturation steps to achieve desired affinity and specify objectives. We observe that within a single antibody campaign machine learning can produce optimized sequences that are on average and at the extremes better than what they observe during training. As DNA sequencing of panning rounds can be performed at a low cost our methods can be readily applied to new antibody campaigns to discover CDR sequences that improve affinity and specificity using the methods we have described.

We demonstrated that we can combine machine learning models to reject antibodies that bind undesired targets to improve antibody specificity, a key property for therapeutic safety. Importantly, compared to experimental procedures such as counter panning which is specific to an undesired target, multi-objective machine learning models can be readily applied to improve antibody specificity by building upon data from past antibody campaigns.

Our results suggest machine learning will provide efficient strategies for exploring promising subsets of sequence space for antibody design. Conventional affinity maturation methods, when sufficiently powered, improve the affinity of CDR-H3 sequences by randomization. We note that Ens-Grad proposed sequences with two amino acid changes from panning derived sequences which exhibited 1.899 log10 R1-to-R3 enrichment in stringent washing and 2.888 log10 R1-to-R3 enrichment in standard washing (Supplementary Fig. S3C). A brute force search of all possible one and two changes for 6566 seed sequences would require up to 2.193 × 10^8^ sequences, while Ens-Grad explored certain of these changes within a design budget of 5467 sequences. Thus, machine learning offers a powerful tool for focusing design candidates on optimal sequences.

In the presence of adequate training data, we expect that the general optimization framework we outline will be applicable to a wide range of design challenges including the design of DNA aptamers, RNA aptamers, and general protein sequences. We expect that our methods will work unchanged for other CDRs and with data from other affinity platforms such as yeast display (Boder *et al.*, 2012).

## Supplementary Material

btz895_Supplementary_Data
